# Cytokine profiles of cord and adult blood leukocytes: differences in expression are due to differences in expression and activation of transcription factors

**DOI:** 10.1186/1471-2172-8-18

**Published:** 2007-08-31

**Authors:** Andreas Nitsche, Meixia Zhang, Theresa Clauss, Wolfgang Siegert, Kay Brune, Andreas Pahl

**Affiliations:** 1 Department of Experimental and Clinical Pharmacology and Toxicology, University of Erlangen-Nürnberg, Fahrstr. 17, D-91054 Erlangen, Germany; 2 Charité – CCM, CC14 Haematology/Oncology, Charitéplatz 1, 10117 Berlin, Germany; 3 Robert Koch-Institut, Centre for Biological Safety 1, Nordufer 20, 13353 Berlin, Germany

## Abstract

**Background:**

Stem cell transplantation as therapy for hematological disorders is often hampered by severe graft-versus-host-disease. This may be reduced by umbilical cord blood transplantation, an effect that has been attributed to qualitative differences between neonatal and adult T cells. We compared levels of secreted proteins and cytokine mRNA induced in cord blood leukocytes (CBL) and adult blood leukocytes (ABL) by various stimuli.

**Results:**

While interleukin-2 (IL-2) levels were similar in CBL and ABL, there was less induction of the Th1 cytokine interferon-γ in CBL. Production of the Th2 cytokines IL-4, IL-5, and IL-13 and the hematopoietic cytokine IL-3 was much lower in CBL versus ABL after T-cell receptor-mediated stimulation, whereas production of GM-CSF was comparable in the 2 cell types. The lower levels of Th1 and Th2 cytokines were maintained in CBL during a 4-day time-course study, while after 12 hours IL-3 and GM-CSF reached in CBL levels similar to those in ABL. For all cytokines except IFNγ, the IC_50 _values for inhibition by cyclosporin A were similar in ABL and CBL. In contrast, there was less expression and activation of transcription factors in CBL. Activation of NF-κB by TPA/ionomycin was detected in ABL but not CBL. Furthermore, there was less expression of the Th subset-specific transcription factors T-bet and c-maf in CBL versus ABL, whereas GATA-3 expression was similar. Expression of T-bet and c-maf correlated with expression of the Th1 and Th2 cytokines, respectively. Time course experiments revealed that T-bet expression was stimulated in both cell types, whereas c-maf and GATA-3 were induced only in ABL.

**Conclusion:**

The diminished capability of CBL to synthesize cytokines is probably due to decreased activation of NF-κB, whereas differences in Th subsets are due to differences in regulation of Th lineage-specific transcriptions factors. We propose that the reduced incidence and severity of GvHD after allogeneic transplantation of umbilical CB cells is due to lesser activation of specific transcription factors and a subsequent reduction in production of certain cytokines.

## Background

Allogeneic stem cell transplantation (SCT) is an accepted treatment for a variety of benign and malignant hematological disorders [1]. Successful SCT requires suitable donors. These may be siblings, other family members, or human leukocyte antigen (HLA)-matched unrelated individuals. Due to the complexity of the HLA system, donors cannot be found for all patients needing a transplant. Close matching confers a higher probability of successful engraftment and minimizes the risk of potentially fatal graft-versus-host disease. Unfortunately, there is only a 25% chance for identifying a full HLA match in a sibling donor[2]. Therefore, interest has developed in potential new sources of stem cells such as cord blood (CB), which is rich in hematopoietic stem cells and precursor cells. First experiences with this new stem cell source for clinical transplantation showed that it can be used even in the presence of major HLA disparities between recipients and related or unrelated donors [3]. Despite major HLA differences, the graft failure rate and incidence and severity of graft-versus-host disease (GvHD) are low [3,4]. The reasons for this are still unknown. It is speculated that GvHD is less severe because CB recipients receive fewer T cells than do recipients of bone marrow or peripheral blood (PB) stem cells and that cord blood lymphocytes (CBL) are more immature and not yet sensitized to HLA antigens [5]. However, there may be inherent differences in the composition and functional activity of lymphocyte subsets, and in antigen-presenting cells in CB and adult blood (AB) [5,6]. The incidence and severity of GvHD may be sensitive to not only factors relevant to transplantation such as HLA compatibility, donor and recipient age, T-cell inoculum, and the source of stem cells but also to the effects of a number of cytokines. Therefore, it is important to investigate whether differences in cytokine production between CB and AB can account in part for the reduced incidence of graft versus host disease with CB transplantation.

A variety of experimental techniques, including enzyme-linked immunosorbent assay (ELISA) and bioassays, have been used to measure cytokine secretion by CB cells and AB cells. Unfortunately, these studies have produced limited and inconclusive results [7,8]. Reduced production of intracellular interleukin-2 (IL-2), IL-4, tumor necrosis factor-α (TNF-α), and interferon-γ (IFN-γ) was detected by flow cytometry in phorbol 12-myristate 13-acetate-activated and ionomycin-activated CBL compared with ABL [9]. ELISA demonstrated that purified CD4^+^CD45RA^+ ^cells from CB produce only 10% of the amount of IL-2 produced by cells from AB. It was hypothesized that this may play a crucial role in the reduced development of GvHD with CB transplantation [10].

To overcome the inherent problems of previously used techniques we investigated cytokine production in CB and AB using real-time RT-PCR. We quantified mRNA expression and protein levels of a number of Th1, Th2, and hematopoietic cytokines synthesized in response to a variety of T-cell stimuli. In addition, we quantitatively analyzed the activation and expression of relevant T-cell transcription factors.

## Results

### Analysis of cell populations

Consistent with previous reports, there were higher total leukocyte counts in CB versus AB [9]. Total leukocyte counts ranged from 5,200 to 9,800/mm^3 ^in ABL and from 11,400 to 22,700/mm^3 ^in CBL. We also determined the numbers of cells expressing CD3, CD4, CD28, and CD45 in mononuclear cell preparations from AB and CB (Table 1). There were a slightly higher number of CD3^+ ^cells in ABL than CBL, whereas the numbers of CD4^+ ^cells were similar. Double-staining showed that all CD4^+ ^cells were also CD3^+^. CBL had a smaller proportion of CD3^+ ^cells that were not CD4^+ ^than did ABL, indicating lower numbers of CD8^+ ^cells in CBL. CD28 was similarly expressed in CBL and ABL. Almost all CD3^+ ^cells were also CD28^+^. Consistent with previous reports, CBL contained very few CD45RO^+ ^cells; there were greater numbers in ABL. There were similar numbers of CD45RA^+ ^cells in ABL and CBL.

**Table 1:** Distribution of surface antigens on CBL and ABL

% Positive	CD3^+^	CD4^+^	CD4^+^/CD3^+^	CD28^+^	CD3^+^/CD28^+^	CD45RA^+^	CD45RO^+^
CBL	25.6 ± 2.9	18.7 ± .2	100.0 ± 0.1	24.7 ± 2.8	96.6 ± 1.8	41.3 ± 5.2	1.1 ± 0.3
ABL	30.1 ± 4.1	17.8 ± 2.2	99.7 ± 0.5	27.8 ± 4.2	91.3 ± 4.6*	45.3 ± 3.6	11.6 ± 2.8**

Mononuclear cells were isolated from whole blood by density gradient centrifugation over Histopaque 1077. The numbers of positive cells were determined by 1 or 2-color FACS analysis and are expressed as the percent of positive cells among total leukocytes. Data are mean ± standard error for 9 donors. *p < 0.05 ABL vs. CBL. **p < 0.01 ABL vs. CBL.

### CBL and ABL cytokine responses to stimuli

Since CBL and ABL similarly expressed the cell surface receptors CD3 and CD28, which transduce activation signals from the cell surface to the interior, we analyzed the responses of CBL and ABL to different stimuli. Mitogenic substances and antibodies stimulating surface receptors were used in different combinations, and the induction of various cytokines was measured by quantitative real-time RT-PCR and ELISA. TPA and ionomycin were chosen because they act synergistically and are able to bypasses the requirement for antigen- or lectin-induced signal at the onset of lymphocyte activation [11]. CD3- and CD28-antibodies were chosen, because they are able to mimick the two signals needed for antigen specific avtivation of T-Cells.

### Th1 cytokines

**Figure 1:**
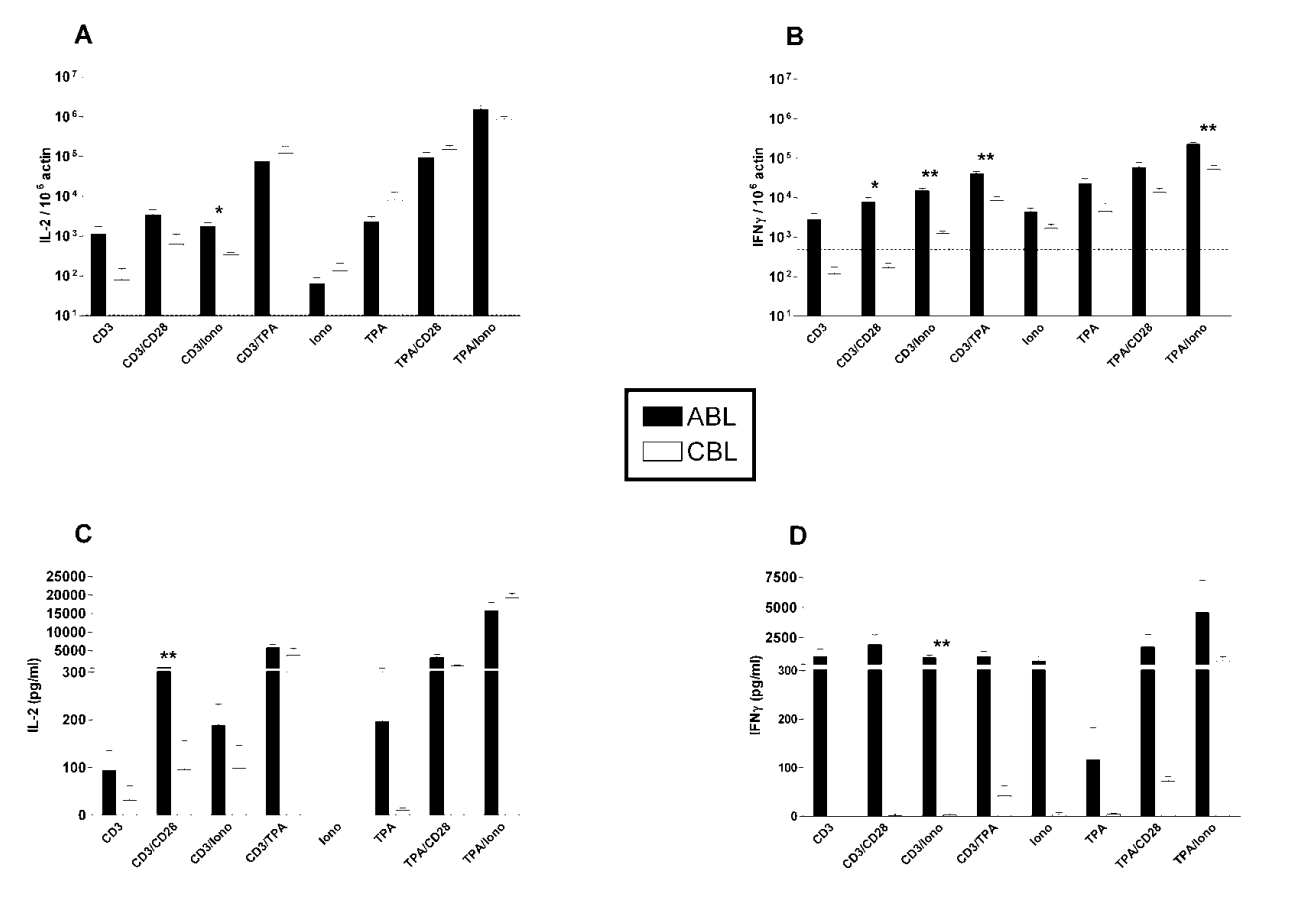
Induction of Th1 cytokine mRNA and protein in ABL and CBL. mRNA levels (A, B) were determined using real-time RT-PCR after 4 hrs of stimulation. Data are expressed as arbitrary units normalized to β-actin to correct for RNA quantity and integrity. The dotted line indicates the basal mRNA level of untreated cells. Protein levels (C, D) were determined in the supernatant by ELISA after 24 hrs of stimulation. Bars are mean ± standard error for 3 donors. Similar results were obtained in 3 independent experiments. Although there was a clear coinciding tendency in all experiments, individual differences between blood samples often reduced the significance of the differences between CB and AB. Iono – Ionomycin. *p < 0.05, **p < 0.01 ABL vs. CBL.

For the Th1 cytokines IL-2 and IFNγ a combination of the mitogenic stimuli TPA and ionomycin caused the highest induction of mRNA and protein in both ABL and CBL (Fig 1). These mitogenic stimuli induced similar levels of IL-2 mRNA and protein in ABL and CBL. In contrast, stimulation of cell surface receptors was approximately 10-fold less effective in inducing expression of Th1 cytokines in CBL versus ABL. Only with combinations including at least one mitogenic stimulus Th1 cytokine levels were raised to a similar degree in CBL as in ABL. This difference was most pronounced for IFNγ; regardless of the stimulus applied, CBL produced less IFNγ protein. In CBL, IFNγ protein was induced only by combined stimulation with TPA and ionomycin, and at far lower levels than in ABL.

### Th2 cytokines

**Figure 2:**
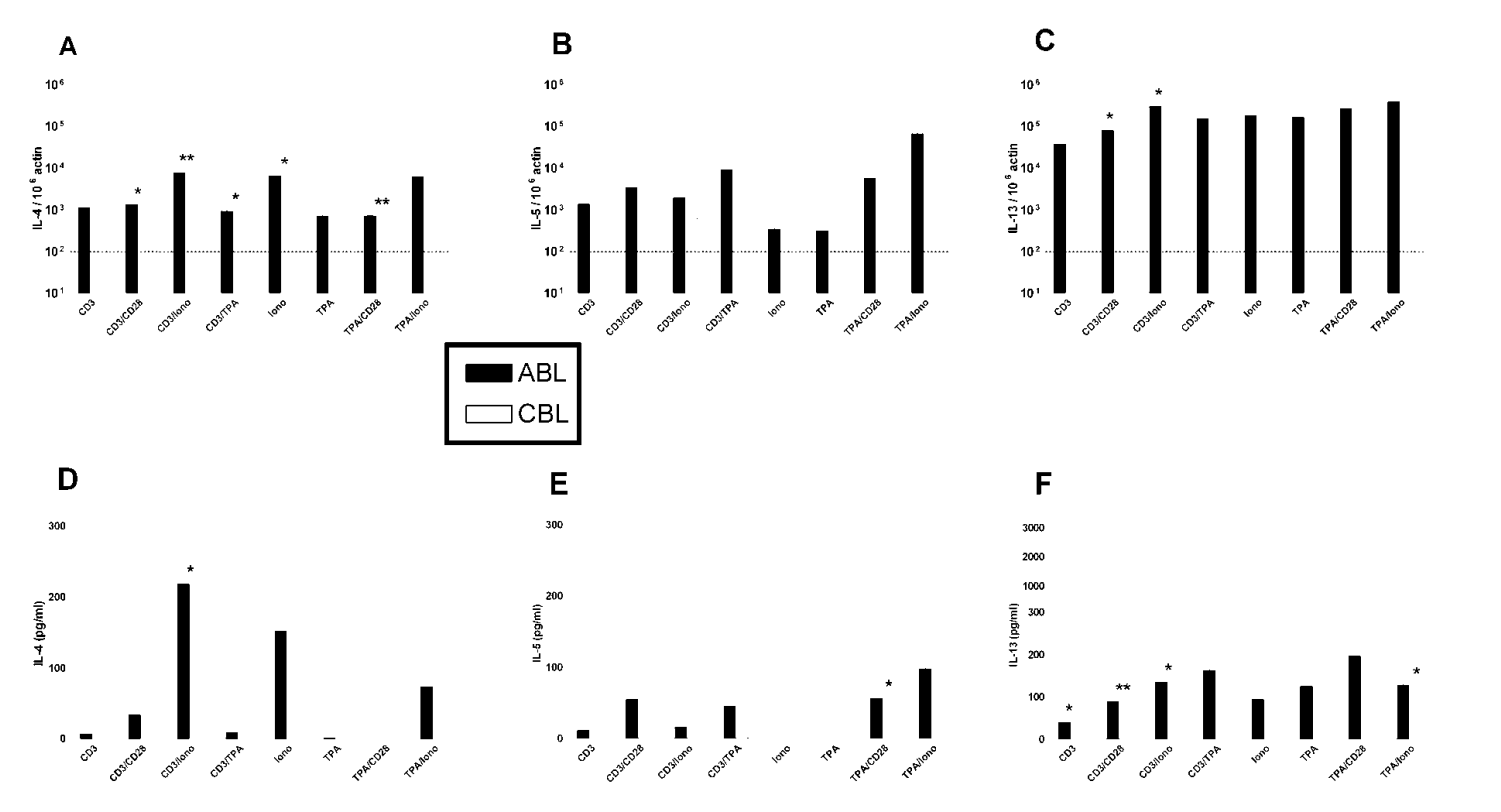
Induction of Th2 cytokine mRNA and protein in ABL and CBL. mRNA levels (A, B, C) were determined using real-time RT-PCR after 4 hrs of stimulation. Data are expressed as arbitrary units normalized to β-actin to correct for RNA quantity and integrity. The dotted line indicates the basal mRNA level of untreated cells. Protein levels (D, E, F) were determined in the supernatant by ELISA after 24 hrs of stimulation. Bars are the mean ± standard error for 3 donors. Similar results were obtained in 3 independent experiments. Iono – Ionomycin. *p < 0.05, **p < 0.01 ABL vs. CBL.

Induction of the IL-4, IL-5, and IL-13 was assessed by real-time RT-PCR and ELISA, as described above (Fig 2). Highest induction of IL-4 in both cell types was observed, when ionomycin was present as stimulus. However, the levels of IL-4 were lower in CBL than in ABL. α-CD3 and α-CD28 induced moderately IL-4 mRNA and protein in ABL but were rather inefficient in CBL. IL-5 mRNA level were close to the dectection limit and IL-5 protein was barely detectable in CBL, whereas a combination of any two stimuli induced detectable amounts of IL-5 in ABL. In CBL, IL-13 mRNA and protein were induced only with the application of at least one mitogenic stimulus, whereas in ABL there was little difference in the level of IL-13 induction with different stimuli. However, similar or even higher levels of IL-13 mRNA and protein were detected in CBL compared to ABL.

### Hematopoietic cytokines

**Figure 3:**
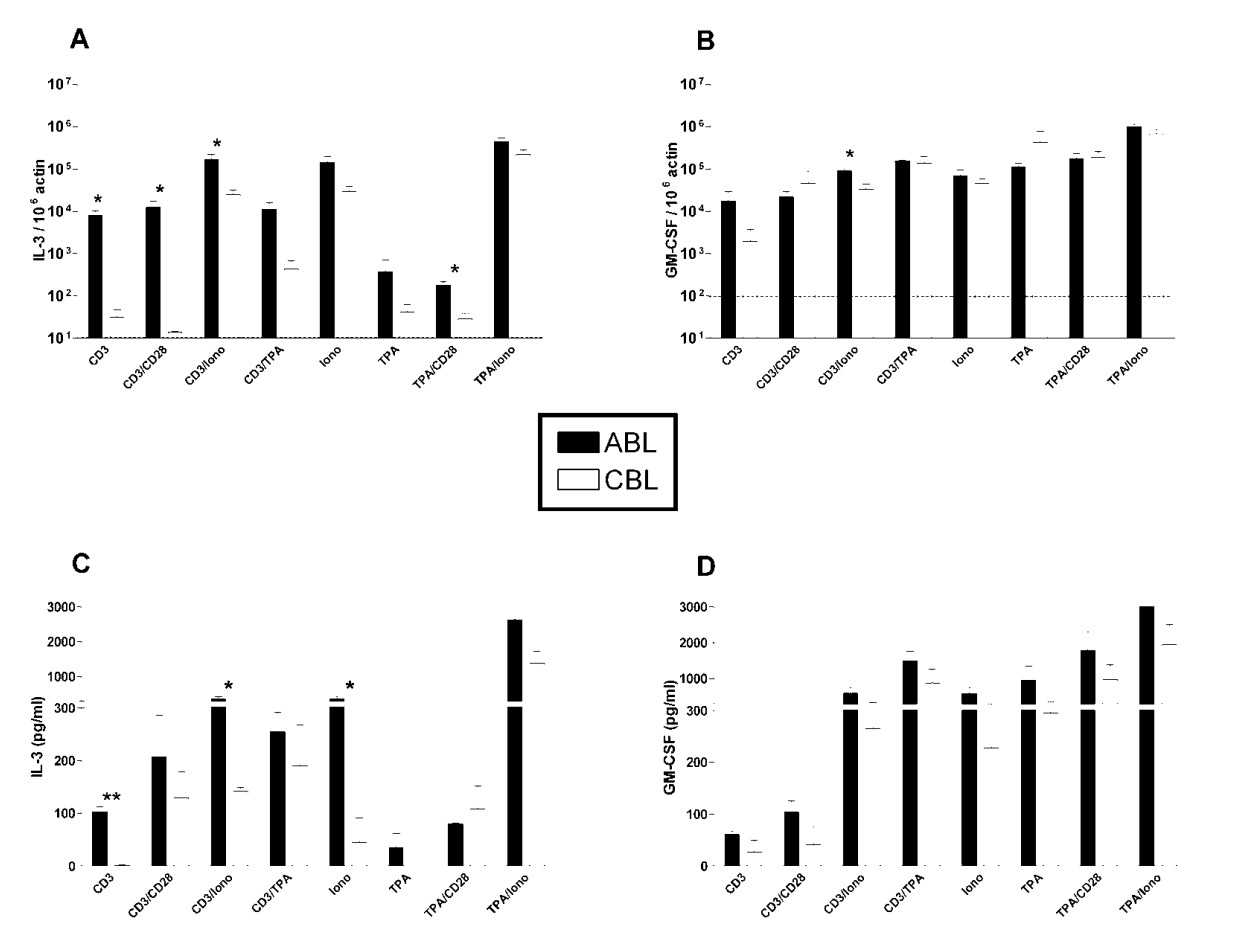
Induction of hematopoietic cytokine mRNA and protein in ABL and CBL. mRNA levels (A, B) were determined using real-time RT-PCR after 4 hrs of stimulation. Data are expressed as arbitrary units normalized to β-actin to correct for RNA quantity and integrity. The dotted line indicates the basal mRNA level of untreated cells. Protein levels (C, D) were determined in the supernatant by ELISA after 24 hrs of stimulation. Bars are the mean ± standard error for 3 donors. Similar results were obtained in 3 independent experiments. Iono – Ionomycin. *p < 0.05, **p < 0.01 ABL vs. CBL.

CB and AB differ in their poietic and bone marrow repopulating capacities [12]. Therefore, we evaluated CBL and ABL production of the hematopoietic cytokines IL-3 and GM-CSF (Fig 3). The strongest induction of IL-3 mRNA and protein occurred in the presence of ionomycin. α-CD3 and α-CD28 more potently stimulated IL-3 mRNA in ABL versus CBL. GM-CSF was the only cytokine for which there were no significant differences in mRNA or protein between ABL and CBL. Mitogenic stimuli induced much higher levels of GM-CSF protein than did stimulation of surface receptors alone.

### Kinetics of cytokine production

**Figure 4:**
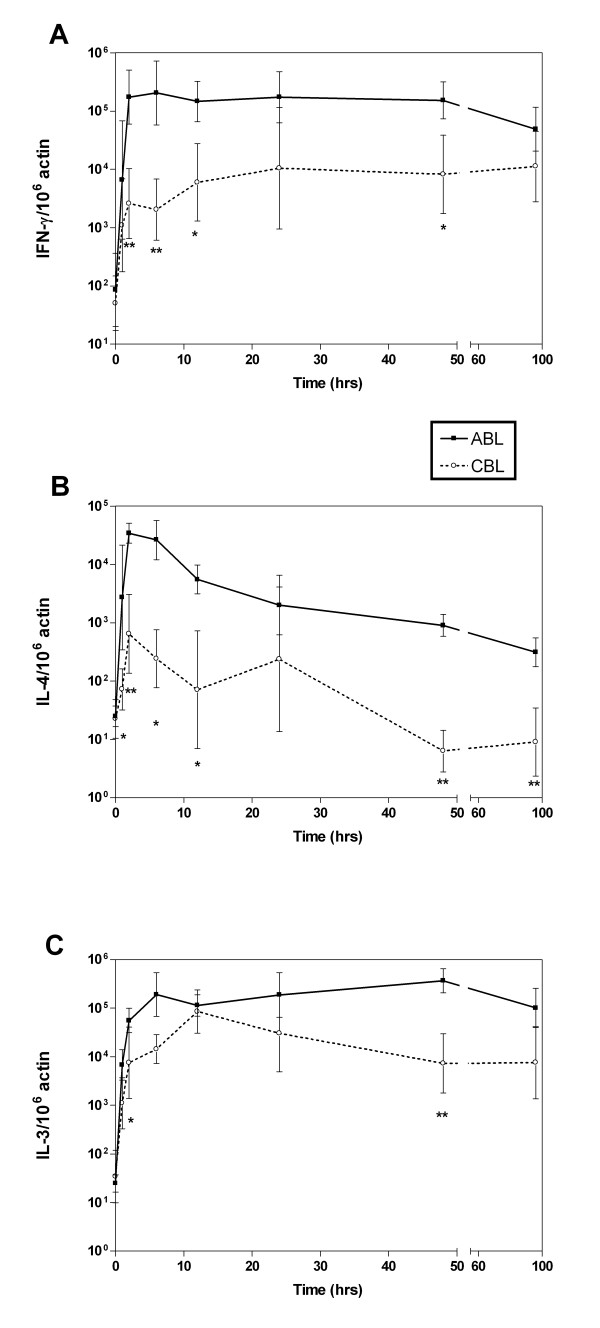
Kinetics of cytokine induction after stimulation with TPA and ionomycin. ABL and CBL were harvested at different time points by lysing cells. mRNA levels for IFNγ (A), IL-4 (B), and IL-3 (C) were determined as in Figure 1. Data points are the mean ± standard error for 6 donors. *p < 0.05, **p < 0.01 ABL vs. CBL.

Differences in cytokine expression between ABL and CBL may stem from different induction kinetics. Therefore, we analyzed levels of cytokine mRNA at a series of time points up to 4 days after stimulation. Since the combination of TPA and ionomycin induced detectable levels of all cytokines, regardless of differences in receptor expression, we focused on this stimulus. Because the kinetics were similar within each group of cytokines (Th1, Th2, and hematopoietic cytokines), only data from one cytokine in each group is shown in Figure 4. mRNA levels of the Th1 cytokines IL-2 and IFNγ peaked 6 hrs after stimulation in ABL and 24 hrs after stimulation in CBL (Fig 4A). Levels remained nearly constant for the remainder of the observation period. At each time point mRNA expression was approximately one order of magnitude greater in ABL versus CBL. This difference was significant at 2,6, 12 and 48 hours. On day 4, mRNA levels declined in ABL, whereas levels increased slightly in CBL reaching similar levels as in ABL.

In contrast to these patterns, mRNA levels for the Th2 cytokines IL-4, IL-5, and IL-13 peaked between 2–6 hrs then steadily declined (Fig 4B). The levels of IL-4 and IL-5 expression were approximately 2 orders of magnitude higher in ABL versus CBL, and the level of IL-13 expression was approximately one order of magnitude higher in ABL versus CBL. This difference was significant at evey time point except 24 hours.

Patterns of expression of the hematopoietic cytokines IL-3 and GM-CSF were remarkably different. In CBL, the kinetics of IL-3 and GM-CSF expression were delayed; mRNA levels peaked at 12 hrs (Fig 4C). In contrast, in ABL, after an initial increase in mRNA levels between 0 and 6 hrs, levels were reduced at 12 hrs but thereafter increased again, peaking after 48 hrs. IL-3 levels in ABL and CBL were similar after 12 hrs but differed before and after this time point. Remarkably, this is the only time point at which levels of expression of any of the cytokines were comparable for CBL and ABL. IL-3 levels were only significantly different at 2 and 48 hours.

### Sensitivity of ABL and CBL to CsA

CsA is a potent inhibitor of various T-cell-derived cytokines. It is an effective prophylactic and treatment for GvHD. Therefore, we investigated possible difference in dose-responsiveness of ABL and CBL to CsA. Cells were preincubated with different concentrations of CsA and subsequently stimulated with a combination of TPA and ionomycin. Table 2 shows the IC50 of production of cytokine protein after a 24-hr stimulation period. Results from mRNA expression after a 6-hr stimulation period were comparable to the IC50s for cytokine protein inhibition after 24 hours (data not shown). All cytokines were sensitive to inhibition by CsA in both ABL and CBL. The IC50 values ranged between 3 and 10 nM and did not differ significantly between cytokines or cell sources.

**Table 2:** IC_50_ values for the inhibition of cytokine protein synthesis by CsA in ABL and CBL

Cytokine	CBL (nM)	ABL (nM)
IL-2	4.52 ± 0.70	3.36 ± 0.13
IFNγ	4.32 ± 0.89	12.60 ± 5.05
IL-4	3.43 ± 0.68	3.71 ± 0.16
IL-5	3.27 ± 0.91	4.73 ± 1.23
IL-13	4.42 ± 0.67	3.74 ± 0.06
IL-3	3.20 ± 0.41	5.56 ± 1.03
GM-CSF	7.75 ± 0.53	9.02 ± 0.44

Cells were stimulated by TPA/ionomycin for 24 hrs. Protein levels were determined in the supernatant by ELISA. Data shown are mean IC_50 _± standard error for 3 donors.

### Activation of transcription factors in ABL and CBL

**Figure 5:**
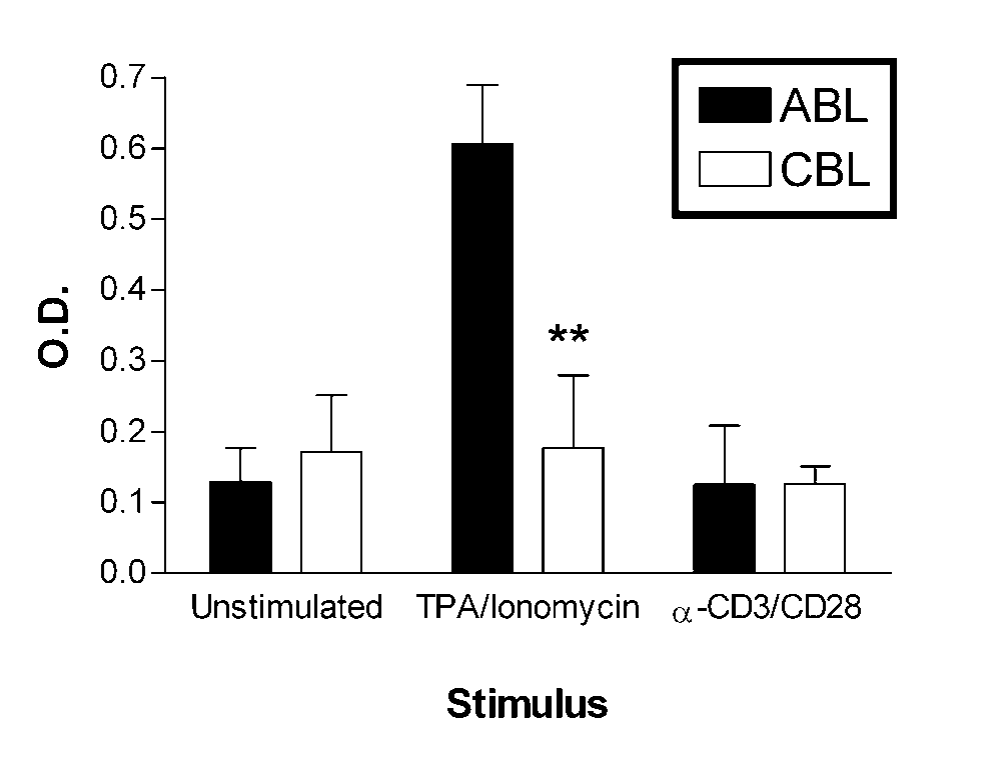
Activity of transcription factors. Nuclear extracts were prepared 60 min after stimulation. NF-κB p50 DNA binding activity was determined by ELISA as described in the Materials and Methods. The optical density (O.D.) was detected at 655 nm. Each bar represents the mean ± standard error for 3 donors. **p < 0.01 ABL vs. CBL.

Since there was reduced cytokine protein and mRNA expression in CBL, we investigated whether the expression of transcription factors is also reduced in CBL. First, we assessed the activation of the more general transcription factor nuclear factor-kappa B (NF-κB). We determined the DNA-binding activity of NF-κB subunit p50 in nuclear extracts using ELISA of DNA-bound transcription factor molecules. As shown in Figure 5, the basal activity of NF-κB was similar in ABL and CBL. TPA and ionomycin stimulated NF-κB activity in ABL, whereas hardly any increase in activity was detected in CBL. α-CD3/α-CD28 stimulation did not affect the activity of NF-κB in either cell type.

### Expression of transcription factors in ABL and CBL

**Figure 6:**
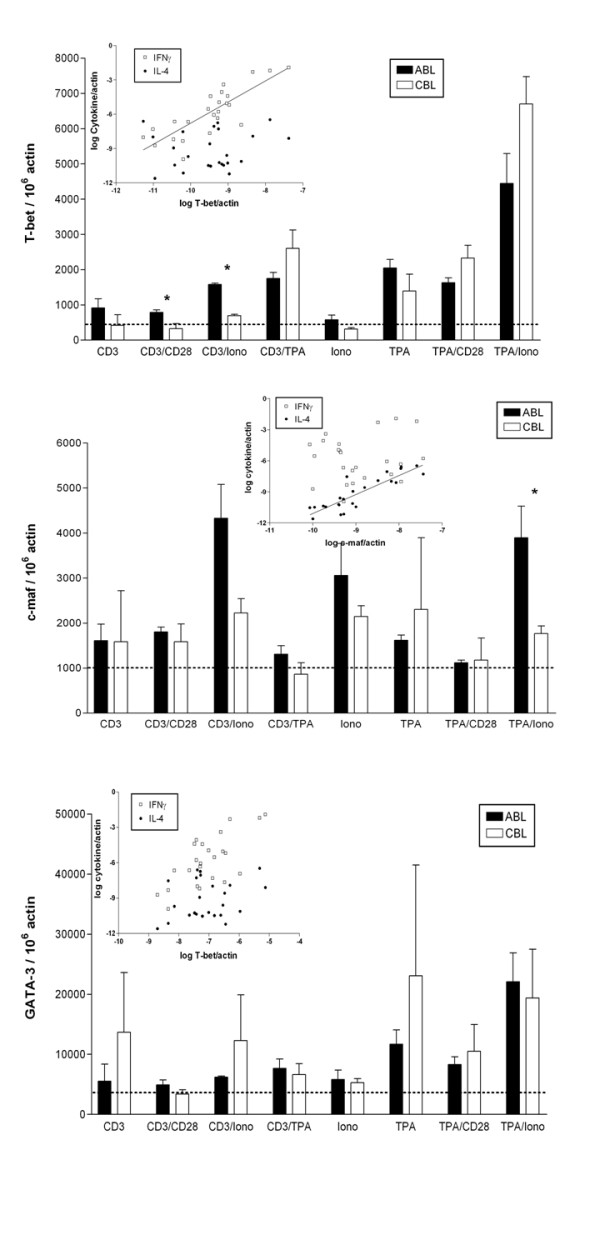
Induction of transcription factors in ABL and CBL after stimulation for 4 hrs. mRNA levels of T-bet (A), GATA-3 (B), and c-maf (C) were determined using real-time RT-PCR. Data are expressed as arbitrary units normalized to β-actin to correct for RNA quantity and integrity. Columns are the mean ± standard for 3 donors. The inset shows the correlation between expression of the respective transcription factor with IFNγ and IL-4. Dotted line indicates basal transcript levels. Iono – Ionomycin.

Since there was deficient production of Th2 cytokines in CBL, we determined whether CBL and ABL differently expressed Th subset-specific transcription factors. Recently, T-bet was described as a Th1-specific transcription factor. Since no antibodies against human T-bet were available at the time of the study, we compared the mRNA expression of T-bet in ABL and CBL exposed to various stimuli (Fig 6A). There was a specific defect in T-bet expression in CBL after antigenic stimulation. Following TPA stimulation, expression levels were similar in ABL and CBL, and the pattern of expression mirrored that for Th1 cytokines (Fig 1). This is shown by the significant correlation between expression of T-bet and IFNγ (*r*^2 ^= 0.68; *p *< 0.0001) but not IL-4 (*r*^2 ^= 0.01) (Fig 6A). In contrast, expression of c-maf, which has been described as a Th2-specific transcription factor, mirrored that of Th2 cytokines (Fig 6B). There was a significant correlation between expression of c-maf and IL-4 (*r*^2 ^= 0.76, *p *< 0.0001), but no significant correlation with IFNγ (*r*^2 ^= 0.02). c-maf expression was induced only by ionomycin only in ABL (Fig 6B). Expression of GATA-3, another Th2-specific transcription factor, did not correlate significantly with IL-4 or IFNγ expression (Fig 6C). There was no significant difference in induction of this transcription factor in CBL versus ABL, suggesting no immediate function of GATA-3 in the defective cytokine production in CBLs (Fig 6C).

**Figure 7:**
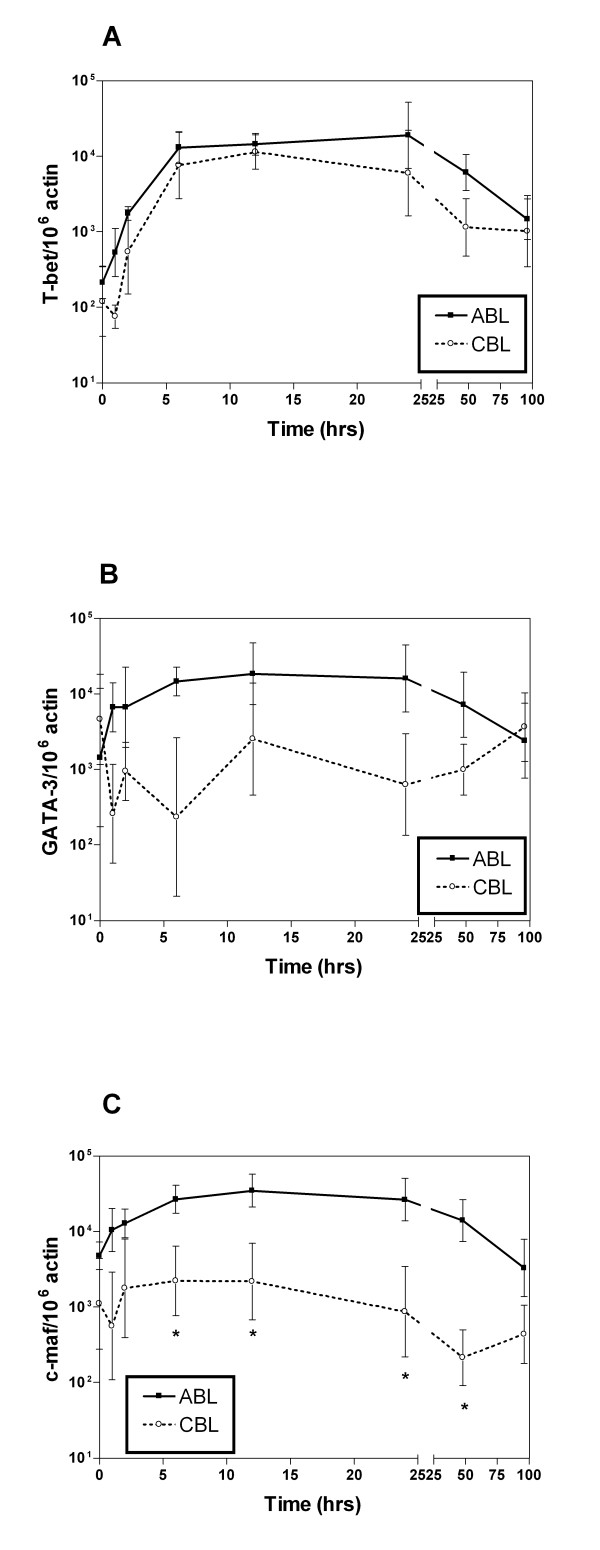
Time course of transcription factor induction after stimulation with TPA and ionomycin. ABL and CBL were harvested at different time points by lysing cells. mRNA levels were determined for T-bet (A), GATA-3 (B), and c-maf (C). Data points are mean ± standard error for 6 donors. *p < 0.05 ABL vs. CBL.

We also studied the time course of expression of transcription factors after TPA/ionomycin stimulation. T-bet expression underwent a similar increase in ABL and CBL during the first 6 hrs and started to decrease after 24 hrs (Fig 7A). Levels were only slightly lower in CBL versus ABL. In contrast, TPA/ionomycin increased the expression of c-maf and GATA-3 only in ABL, whereas levels in CBL remained almost constant (Fig 7B,C). In ABL, the time course of expression was similar to that for T-bet: a rapid increase during the first 6 hrs and a decrease after 1 day.

## Discussion

Because neonates are in a tolerizing status and adults are not, it has been widely assumed that neonatal and adult T cells differ qualitatively. However, recent findings have generated debate regarding the differences between neonatal and adult T cells and whether they are quantitative or due in part to differences in cytokine production. In this study, we examined the induction of cytokine mRNA and protein in human ABL and CBL. Different combinations of mitogenic substances and antibodies stimulating cell surface receptors induced the Th1, Th2, and hematopoietic cytokines to a varying extent. Although there were lower cytokine levels in CBL, they were not accompanied by resistance to the immunosuppressive compound CsA, commonly used in bone marrow transplantation. Differences in the ability of ABL and CBL to produce cytokines are likely due to reduced activation of transcription factors. Activation of NF-κB was greater in ABL versus CBL. Expression of T-bet and c-maf correlated with expression of the Th1 and Th2 cytokines, respectively. Time course experiments revealed that T-bet expression was stimulated in both cell types, whereas c-maf and GATA-3 were induced only in ABL.

Comparison of cytokine production 24 hrs after stimulation revealed comparable induction of IL-2 in ABL and CBL. In contrast, induction of the prototypic Th1 cytokine IFNγ was much weaker in CBL, regardless of the stimulus. Even bypassing the T-cell receptor with mitogenic stimuli failed to induce similar levels of IFNγ in CBL and ABL.

The optimal induction stimuli differed for the various Th2 cytokines. IL-4 was efficiently induced only in the presence of ionomycin. The importance of this stimulus for IL-4 was demonstrated previously [13,14]. IL-5 was induced only in the presence of two stimuli. Following most stimuli, IL-4 and IL-5 protein were not detectable in CBL. In contrast, in the presence of mitogenic stimuli, IL-13 reached levels in CBL that were comparable to those in ABL.

It was previously reported that Th2 cells outnumber Th1 cells in neonates [15,16]. However, we found lower expression of both Th1 and Th2 cytokines in CBL versus ABL and no changes in the relation of Th1 and Th2 cytokines between ABL and CBL. Other studies used intracellular staining of cytokines to detect differences between ABL and CBL after stimulation with TPA/ionomycin. In agreement with our data, these studies reported similar levels of intracellular IL-2 in the two cell types and lower levels of IFNγ and IL-4 in CBL versus ABL [9,17]. Thus, the imbalance of these T cell subsets cannot be ascribed solely to levels of cytokine production.

IL-3 expression was greater in ABL than CBL, regardless of the stimulus. Interestingly, GM-CSF reached similar levels in ABL and CBL with most stimuli applied. Surface receptor stimulation remained ineffective, whereas the presence of mitogens efficiently induced this cytokine. Although neither a precise mechanism nor a physiological influence of IL-3 on early hematopoietic processes is known, several lines of evidence suggest that IL-3 inhibits T and B lymphopoietic events and therefore should lead to reduced appearance of GvHD [18]. This hypothesis conflicts with our finding of reduced IL-3 production in CBL. However, there is some *in vivo *and *in vitro *evidence that underlines the limited proliferation and differentiation activities of IL-3, compatible with a reduced potential to repopulate stem cells. In this context, reduced production of IL-3 may be beneficial for stem cell engraftment and bone marrow repopulation in CBL transplant patients.

Since our first series of experiments investigated only 1 time point, we investigated the possibility that differences in the capability of ABL and CBL to produce various cytokines are due to differences in induction kinetics. MRNA levels for all cytokines remained at least 10-fold lower in CBL during the whole period. The only exception is IL-3, whose level of expression remained similar in CBL and ABL after 12 hrs. Chalmers et al. used intracellular staining and flow cytometry to analyze the number of cells producing IL-2, IL4, and IFNγ during a time period of 24 hrs. They found similar numbers of cells producing IL-2 in ABL and CBL, but smaller numbers of cells producing IL-4 and IFNγ in CBL [9].

The lower cytokine levels in CBLs did not lead to drug resistance against the immunosuppressive CsA, which is commonly used in bone marrow transplantation. All cytokines were sensitive to inhibition by CsA, with similar IC_50 _values for ABL and CBL. Also, no difference between protein and mRNA levels was found. An IC_50 _of 10 nM was reported for IL-2 in ABL [19]. McDouall et al. reported greater sensitivity of IL-2-dependent cell proliferation to CsA in CBL [20]. However, they found only minor differences at low CsA concentrations. Furthermore, another study did not find any significant difference in the sensitivity of T-cell effector functions to CsA [21]. Overall, these findings suggest that the benefits of CBL for bone marrow transplantation are probably not due to increased drug resistance to CsA.

Since we found no difference in expression of the CD3 and CD28 receptor molecules in CBL, we investigated whether differences in cytokine expression of ABL and CBL are due to differences in the expression or activation of transcription factors. We showed for the first time that the overall reduced cytokine synthesis in CBL can be explained at least in part to lesser activation of the transcription factor NF-κB. The importance of this transcription factor to the activation of a number of T cell cytokines such as IL-2, IL-4, IFNγ, IL-3, and GM-CSF has been previously described [22]. Similarly, it has been shown that expression of NFAT1 protein is significantly lower in CB T cells versus adult T cells and is only partially expressed after prolonged primary T-cell receptor stimulation [23]. The lack of activation of NF-κB by CD3/CD28 stimulation can be explained by a slower kinetic of NF-κB activation as compared to TPA/ionomycin stimulation [24].

T-bet has been described as a Th1-inducing transcription factor that activates expression of IFNγ [25], whereas c-maf is responsible for tissue-specific expression of the Th2 cytokine IL-4 [26]. We found that the expression of these Th subset-specific transcription factors was regulated by a variety of stimuli. TPA/ionomycin evoked a tremendous upregulation of T-bet. Furthermore, expression of T-bet was higher in ABL versus CBL. Only mitogenic stimuli induced comparable levels in CBL. This pattern mirrors the expression of Th1 cytokines such as IFNγ, as corroborated by the significant correlation between expression of T-bet and IFNγ. In contrast, the Th2 subset-specific transcription factor c-maf was regulated mainly by ionomycin. Ionomycin was effective only in ABL, suggesting a specific defect in calcium signaling in CBL, which may be responsible for the lower expression of Th2 cytokines. We found a significant correlation between the expression of c-maf and IL-4 but not IFNγ. Ionomycin's ability to effectively stimulate IL-4 has been described previously [13]. The expression of another Th2-specific transcription factor, GATA-3, does not seem to be regulated.

T_H_-cell differentiation is found to be mediated by lineage-specific transcription mechanisms. IL-12 regulates T_H_1-cell differentiation by activating the transcription factor signal transducer and activator of transcription 4 (STAT4)[27]. Signalling cascades induced by TCR crosslinking and IL-12 eventually lead to expression of the transcription factor T-bet, which is a master regulator of T_H_1-cell differentiation because it potentiates the production of IFN γ and suppresses the expression of T_H_2 cytokines[27]. By contrast, IL-4 drives T_H_2-cell differentiation through the action of STAT6[28], which upregulates expression of GATA-binding protein 3 (GATA3), a master regulator of T_H_2-cell differentiation that is both necessary and sufficient for T_H_2-cell development[29]. In addition, c-maf, which was identified as the first T_H_2-cell-specific transcription factor that binds the Il4 proximal promoter, has an important role in IL-4 production once the T_H_2-cell programme has been established[30]. Altogether we propose that the differences in cytokine production by Th1 and Th2 subsets can be explained by differences in the expression of the subset-specific transcription factors T-bet and c-maf. However, alternative explanations such as differences in the expression of signal transduction molecules do exist.

Time-course study of the expression of all transcription factors after stimulation by TPA/ionomycin revealed remarkable differences. Expression of T-bet was similar in ABL and CBL. In contrast, c-maf and GATA-3 were only induced in ABL, whereas the expression levels remained nearly constant in CBL. This observation is consistent with the results of the analysis of cytokine production, where the difference in cytokine levels between CBL and ABL was much greater for Th2 cytokines versus Th1 cytokines. This correlation corroborates our hypothesis that the different expression of Th subset specific transcription factors may be responsible for the different cytokine expression level in ABL and CBL.

## Conclusion

This study investigated differences in cytokine production by ABL and CBL and found a generally reduced capability of CBL to produce cytokines. Because it is widely accepted that T-cell mediated cytokine production plays a crucial role in GvHD, we propose that reduced cytokine production in CBL may contribute to a lower occurrence of GvHD. This effect is due not to differences in populations of memory and naive T cells, but may be due to lesser activation of transcription factor NF-κB. Differences in cytokine production by Th1 and Th2 subsets can be explained by differences in the expression of the subset-specific transcription factors T-bet and c-maf. Since no differences in CsA sensitivity were found, it appears that the reduced incidence and severity of GvHD after allogeneic transplantation of umbilical CB cells is due to lesser activation of specific transcription factors and a subsequent reduction in production of certain cytokines.

## Methods

### Materials

Oligonucleotides were synthesized by TIB Molbiol (Berlin, Germany). DMSO, 2-O-tetradecanoylphorbol-13-acetate (TPA), ionomycin, and Histopaque-1077 were purchased from Sigma (Deisenhofen, Germany). Cyclosporin A (CsA) was purchased from CALBIOCHEM (San Diego, CA, USA). Purified anti-human CD3 and purified anti-human CD28 were obtained from PharMingen Becton Dickinson Company (Heidelberg, Germany). RPMI 1640 medium was purchased from Life Technologies (Paisley Scotland). Unless otherwise indicated, all other chemicals were purchased from Sigma Chemical Company.

### Cells

Heparinized peripheral blood samples were obtained from healthy volunteers (9 men and 7 women ranging in age from 22 to 55 years without medication for at least 14 days). Cord blood was obtained from the umbilical vein immediately after vaginal delivery of uncomplicated pregnancies. This study was approved by the local ethics committee. Mononuclear cells were isolated by density gradient centrifugation over Histopaque 1077 (Sigma, Deisenhofen, Germany), washed twice in RPMI 1640 (Life Technologies), and resuspended in medium supplemented with 10% fetal calf serum (Life Technologies) as described previously [31].

For study of cytokine production, ABL or CBL were resuspended at 10^6 ^cells/ml and incubated in 500 μl volumes in 24-well tissue culture plates (Falcon Becton Dickinson Labware) at 37°C with 5% CO_2_. Cells were stimulated with soluble anti-CD3 monoclonal antibody (1 μg/ml), anti-CD28 monoclonal antibody (0.3 μg/ml), TPA (25 ng/ml), ionomycin (1 μM), or combinations thereof [31]. At the times indicated, cells were sedimented by centrifugation. The supernatants were harvested and kept frozen at -80°C until determination of cytokine protein. The cells were lysed by RLT lysis Buffer (QIAGEN) and frozen at -80°C until RNA isolation. ELISA was performed as described by the manufacturer (Pharmingen, Heidelberg, Germany).

### Monoclonal antibodies and flow cytometry

ABL and CBL were stained according to standard procedures. Briefly, 5 × 10^5 ^cells were incubated in 200 μl FACS buffer (1× PBS containing 0.5% fetal calf serum, 0.1% NaN_3_, and 0.1 M glucose) with the respective monoclonal antibodies for 30 min at room temperature. Subsequently, cells were washed twice with FACS buffer and subjected to phenotypic analysis in a flow cytometer (FACScan, Becton Dickinson). A minimum of 10,000 lymphocyte-gated events were acquired and analyzed with CellQuest 4.0 software. FITC- or PE-conjugated monoclonal anti-CD3, anti-CD4, anti-CD28, anti-CD45RA, anti-CD14, anti-CD19, and anti-CD45RO antibodies were used (Pharmingen, Heidelberg, Germany).

### Quantitation of mRNA expression

RNA was prepared from frozen lysates using RNeasy from QIAGEN (Hilden, Germany). One-tube RT-PCR was performed using the TaqMan EZ RT-PCR kit from PE Applied Biosystems (Weiterstadt, Germany). Expression of cytokines was determined in relation to the expression of β-actin by real-time PCR using a TaqMan assay on an ABI Prism 7700. Primers and probes are listed in Table 3. For each RT-PCR, the threshold cycle (C_T_) was determined, defined as the cycle at which the fluorescence exceeds 10 times the standard deviation of the mean baseline emission during cycles 3–10. Cytokine mRNA levels were normalized to the housekeeping gene β-actin according to the following formula: ΔC_T _= C_T_^β-actin ^- C_T_^cytokine^. Results are presented as 2^ΔCT ^based on the results of control experiments yielding a PCR reaction efficiency of approximately 100%.

**Table 3:** Primer and probes used for real-time RT-PCR

Gene	Forward-Primer	Reverse-Primer	TaqMan-Probe
Act	CAGCGGAACCGCTACTTGCCAATGG	TCACCCACACTGTGCCCATCTACGA	ATGCCCTCCCCCATGCCATCCTGCGT
IL-2	GAATGGAATTAATAATTACAAGAATCCC	TGTTTCAGATCCCTTTAGTTCCAG	ATGCCCAAGAAGGCCACAGAACTG
IL-3	GCTCCCATGACCCAGACAAC	GGCAGACATGGCAGGAGATT	AGCTGGGTTAACTGCTCTAACATGATCGATGAAA
IL-4	CCCCCTCTGTTCTTCCTGCT	AGCCCTGCAGAAGGTTTCCT	TGCCGGCAACTTTGTCCACGG
IL-5	AGGATGCTTCTGCATTTGA	TTCTATTATCCACTCGGTGTTC	TATGCCATCCCCACAGAAATTCCCACA
IL-13	GGAGCTGGTCAACATCACCC	CGTTGATCAGGGATTCCAGG	CCAGAAGGCTCCGCTCTGCAATGGC
IFNγ	CGAGATGACTTCGAAAAGCTGAC	CGCTTCCCTGTTTTAGCTGC	TCCAAGTGATGGCTGAACTGTCGCC
GM-CSF	GCCCTGGGAGCATGTGAAT	GCTCCAGGCGGGTCTGTAG	AGGCCCGGCGTCTCCTGAACCT

### DNA binding activity of transcription factors

The DNA binding activity of transcriptions factors was determined using the Transfactor system from Clontech (Heidelberg, Germany). Nuclear extracts were prepared from cells stimulated for 1 hr according to the manufacturer's protocol.

### Statistical analysis

Data are expressed as mean ± standard error. Differences were statistically analyzed using the unpaired Student's t-test. IC50 values were calculated using PRISM 3.0 (GraphPad Software Inc., San Diego, CA, USA).

## Authors' contributions

AN carried out the molecular studies, participated in the cell studies and drafted the manuscript. MZ carried out ELISA and real-time RT-CPR assays. TC carried out cell isolation and cell culture. WS participated in study design and coordination. KB participated in study design and coordination. AP conceived the study, and participated in its design and coordination. All authors read and approved the final manuscript.
